# Intra-individual variations of organophosphate pesticide metabolite concentrations in repeatedly collected urine samples from pregnant women in Japan

**DOI:** 10.1186/s12199-019-0761-4

**Published:** 2019-01-17

**Authors:** Keisuke Hioki, Yuki Ito, Naoko Oya, Shoji F. Nakayama, Tomohiko Isobe, Takeshi Ebara, Kanemitsu Shibata, Naomi Nishikawa, Kunihiko Nakai, Tomota Kamida, Jun Ueyama, Mayumi Sugiura-Ogasawara, Michihiro Kamijima

**Affiliations:** 1 Department of Occupational and Environmental Health, Nagoya City University Graduate School of Medical Sciences, Nagoya, 467-8601 Japan; 2 Center for Health and Environmental Risk Research, National Institute for Environmental Studies, Tsukuba, 305-8506 Japan; 3 Department of Obstetrics and Gynecology, Nagoya City West Medical Center, Nagoya, 462-8508 Japan; 4 Department of Development and Environmental Medicine, Tohoku University Graduate School of Medicine, Sendai, 980-8575 Japan; 5 Department of Medical Technology, Nagoya University Graduate School of Medicine, Nagoya, 461-8673 Japan; 6 Department of Obstetrics and Gynecology, Nagoya City University Graduate School of Medical Sciences, Nagoya, 467-8601 Japan

**Keywords:** Organophosphate insecticides, Pregnant women, Intraclass correlation coefficients, Reproducibility

## Abstract

**Background:**

Low-dose exposure to organophosphate (OP) insecticides during pregnancy may adversely affect neurodevelopment in children. To evaluate the OP exposure levels, single urine sampling is commonly adopted to measure the levels of dialkylphosphates (DAPs), common OP metabolites. However, the inter-day variations of urinary DAP concentrations within subjects are supposed to be large due to the short biological half-lives of the metabolites, and it is thus considered difficult to accurately assess OP exposure during pregnancy with single sampling. This study aimed to assess intra-individual variations of DAP concentrations and the reproducibility of the exposure dose categorization of OPs according to DAP concentration ranges in pregnant women in Japan.

**Methods:**

Urine samples were collected from 62 non-smoking pregnant women (12–22 weeks of gestation) living in Aichi Prefecture, Japan. First morning void (FMV) and spot urine samples taken between lunch and dinner on the same day were collected on five different days during 2 weeks. The concentrations of DAP and creatinine in urine samples were measured using an ultra performance liquid chromatography with tandem mass spectrometry. Creatinine-adjusted and unadjusted concentrations were used for the intraclass correlation coefficient (ICC) calculations and surrogate category analyses.

**Results:**

For all DAP metabolites, the creatinine-adjusted single ICCs exceeded 0.4, indicating moderate reliability. Overall, ICCs of spot urine samples taken in the afternoon were better than those taken as FMV. Surrogate category analyses showed that participants were categorized accurately into four exposure dose groups according to the quartile points.

**Conclusion:**

This study indicated that a single urine sample taken in the afternoon may be useful in assessing OP exposure as long as the exposure is categorized into quartiles when conducting epidemiological studies in early to mid-pregnant women in Japan.

**Electronic supplementary material:**

The online version of this article (10.1186/s12199-019-0761-4) contains supplementary material, which is available to authorized users.

## Background

Organophosphate (OP) insecticides are the most commonly used pesticides for the protection of crops. Their global sales reached 70 million US dollars in 2014 [1], and the shipment value in Japan in 2012 was more than 20 billion Japanese yen (equivalent to 177 million US dollars) [2]. The widespread use of OP pesticides has resulted in ubiquitous exposure in humans. While high-level exposure to OP pesticides from accidental release or deliberate ingestion causes intoxication by inhibition of acetylcholinesterase, the general population in Japan is not exposed to OPs at such levels [3, 4]. Recently, however, it has been reported that low-dose exposure to OP pesticides may adversely affect human neurodevelopment, particularly if the exposure occurs during the prenatal period when the fetal brain is undergoing rapid development and the ability to detoxify OPs has not yet matured [5]. Potential positive associations have been reported for prenatal OP exposures with lower intelligence quotient [6], increases in attention problems and attention deficit hyperactivity disorder [7], and an increase in abnormal reflexes [8].

To evaluate these potential effects of OPs, single urine sampling, of which the collection procedure is noninvasive and relatively simple, has been commonly adopted to determine the levels of common OP metabolites (dialkylphosphates (DAPs) including dimethylphosphate (DMP), dimethylthiophosphate (DMTP), dimethyldithiophosphate (DMDTP), diethylphosphate (DEP), diethylthiophosphate (DETP), and diethyldithiophosphate (DEDTP)). Nonetheless, there is a concern about the representativeness of the measured DAP concentrations as biomarkers of OP exposure during a certain period. The main issue is that the inter-day variations of urinary DAP concentrations are supposed to be large due to the following two reasons: (a) the general population is exposed to OPs mainly through the diet that changes every day [9] and (b) the biological half-lives of most OPs are within the range of 12–36 h [10], which indicates that they are excreted from the body within hours to days [11].

The intraclass correlation coefficient (ICC) is often used as the indicator of intra-individual variation. There are some studies reporting ICCs for the DAP variability in pregnant women from Europe [12] and Puerto Rico [13], but no information is available from East Asia. The previous studies reported that the ICCs of DAP represented low values, indicating that it is hard to accurately assess OP exposure with a single sample of spot urine. Japan-specific ICC derivation is needed because insecticide use, diets, and race which possibly contribute to DAP concentrations differ from those paid attention to in the previous studies.

Therefore, this study aimed to assess intra-individual variations of DAP and the reproducibility of the exposure dose categorization of OP insecticides according to DAP concentration ranges in pregnant women in Japan.

## Methods

### Subjects and urine sampling

Non-smoking women between 9 and 20 weeks of pregnancy were asked to participate in this study at Nagoya City West Medical Center, Japan. Informed consent was obtained from all participants. In total, 62 recruited women (average age = 33.6 ± 4.8 years) between 12 and 22 weeks of gestation provided urine samples. The samples of first morning void (FMV) and spot urine taken between lunch and dinner on the same day were collected on five different days within consecutive 2 weeks. Both FMV and afternoon spot urine samples were stored in a cold box with a refrigerant after collection and handed to a refrigerated cargo service on the collection day. Samples were kept cold and transported to the National Institute for Environmental Studies. After arrival, urine samples were stored at − 20 °C for a few days before transferring to an − 80 °C storage unit until use. A total of 617 urine samples were collected from the 62 pregnant women, including three who provided nine urine samples.

### Urinary DAPs and creatinine measurements

The concentration of DAP in the samples was measured according to the previously reported method using an ultra performance liquid chromatography with tandem mass spectrometry (UPLC-MS/MS) [14, 15] with slight modifications as follows. At the preparation step in the present study, the concentration of formic acid added to the samples was determined to be 99.9%, instead of 100 mM. Additionally, DEP was measured after eluting it with 2.5% ammonia in 50% (*v*/*v*) acetonitrile/water, which yielded better recovery for solid-phase extraction. The limit of detection (LOD) of DMP, DMTP, DMDTP, DEP, DETP, and DEDTP was 0.06, 0.10, 0.63, 0.47, 0.02, and 0.02 μg/L, respectively, at which signal-to-noise ratios were three. Concentrations below the LOD were regarded as LOD/√2 [16]. Total concentrations (nmol/L) of dimethyl DAP (ΣDMAP; the sum of DMP, DMTP, and DMDTP), diethyl DAP (ΣDEAP; the sum of DEP, DETP, and DEDTP), and DAP (ΣDAP; the sum of ΣDMAP and ΣDEAP) were calculated. Urinary creatinine (Cr) concentrations were also measured by UPLC-MS/MS as described elsewhere [17]. Standard curves for DAPs and Cr were determined using seven different concentrations of each standard-spiked urine sample. DAP- or Cr-spiked standard urine samples, which were divided into 4 ml aliquots stored at − 80 °C until use, were inserted into every 40 samples as quality controls. All blanks were free of detectable DAP contamination. The DAP remained stable in response to temperature changes and freeze-thaw cycles; any changes were negligible [14]. Each batch contained 80 or less samples.

### Statistical analyses

Since Cr excretion into urine is dependent on many exogenous and endogenous factors [18], both Cr-adjusted and unadjusted DAP concentrations were used for ICC calculation, which is described as a ratio of the between-person variance divided by the sum of the between-person and within-person variance, and for surrogate category analyses as mentioned below. The single measure and average measure ICCs which reflect the reliability of the ratings for single observation and average score across a number of observations, respectively, were calculated using a one-way random effects model [19] available in SPSS version 23 (IBM Japan, Tokyo, Japan) on log-transformed DAP concentrations that were originally non-normally distributed. ICCs of the samples obtained as FMV were compared with ICCs of the spot samples obtained in the same afternoon. ICCs were assessed by the criteria proposed by Landis and Koch [20]. ICC values higher than 0.80 were categorized as “almost perfect,” and the ranges of 0.60–0.80, 0.40–0.60, and < 0.40 were categorized as “substantial,” “moderate,” and “fair,” respectively [20]. The number of subjects required per group was also calculated using ICC [21].

Surrogate category analyses [22] were performed to address how much exposure misclassification may occur when participants were categorized into four exposure groups according to the quartile points, i.e., 16 pregnant women for the lowest, 15 pregnant women for the mid-low, 15 pregnant women for the mid-high, and 16 pregnant women for the highest quartile. One thousand reiterations were performed for the sampling and classification steps. If surrogate categories were correctly assigned, the mean value of each category should increase monotonically from the lowest to the highest exposure category, and the percentage of this success rate was calculated.

## Results

The distribution of unadjusted and Cr-adjusted urinary DAP concentrations among pregnant women is summarized in Table 1 and Additional file 1: Table S1, respectively. Geometric means (ranges) and detection rates of urinary DAPs were 1.5 μg/L (< LOD–81) and 87% for DMP, 5.3 μg/L (< LOD–332) and 98% for DMTP, 0.94 μg/L (< LOD–12) and 66% for DMDTP, 3.6 μg/L (< LOD–153) and 99% for DEP, 0.73 μg/L (0.11–102) and 100% for DETP, and 0.05 μg/L (< LOD–2.4) and 67% for DEDTP, respectively. The maximum between-subject difference in ΣDAP was approximately 313-fold (min 8.7 nmol/L, max 2742 nmol/L). Geometric means (ranges) of urinary Cr-adjusted DAPs in FMV and afternoon spot urine were 2.0 μg/g Cr (< LOD–55) and 1.8 μg/g Cr (< LOD–98) for DMP, 8.1 μg/g Cr (< LOD–262) and 6.8 μg/g Cr (< LOD–569) for DMTP, 1.2 μg/g Cr (< LOD–17) and 1.2 μg/g Cr (< LOD–17) for DMDTP, 5.0 μg/g Cr (< LOD–143) and 5.1 μg/g Cr (< LOD–130) for DEP, 1.0 μg/g Cr (0.11–240) and 1.0 μg/g Cr (0.11–207) for DETP, and 0.05 μg/g Cr (< LOD–1.6) and 0.07 μg/g Cr (< LOD–6.9) for DEDTP, respectively (Additional file 1: Table S1). The between-variance was much higher than the within-variance in both unadjusted and Cr-adjusted DAP concentrations.

**Table 1:** Limit of detection, detection rates, geometric means, and percentile values of urinary dialkylphosphate concentrations among pregnant women in Japan

Compounds	LOD	> LOD (%)	GM	Min.	Percentile					Max.	Between-variance	Within-variance
					5th	25th	50th	75th	95th			
DMP	0.06	87	1.5	< LOD	0.09	0.60	1.8	4.7	18	81	14	1.4
DMTP	0.10	98	5.3	< LOD	0.41	2.0	5.6	17	54	332	10	1.5
DMDTP	0.63	66	0.94	< LOD	< LOD	< LOD	0.73	1.7	4.7	12	2.6	0.44
DEP	0.47	99	3.6	< LOD	0.69	1.9	3.4	6.5	21	153	4.8	0.56
DETP	0.02	100	0.73	0.11	0.16	0.29	0.57	1.6	7.17	102	7.4	0.79
DEDTP	0.02	67	0.05	< LOD	< LOD	< LOD	0.05	0.12	0.41	2.4	8.0	0.79
ΣDMAPs	–	–	70	4.0	7.9	29	64	177	535	2621	7.2	0.89
ΣDEAPs	–	–	30	2.9	6.3	15	28	53	178	1005	5.1	0.53
ΣDAP	–	–	110	8.7	18	51	100	249	648	2742	5.9	0.66

*DMP* dimethylphosphate (μg/L); *DMTP* dimethylthiophosphate (μg/L); *DMDTP* dimethyldithiophosphate (μg/L); *DEP* diethylphosphate (μg/L); *DETP* diethylthiophosphate (μg/L); *DEDTP* diethyldithiophosphate (μg/L); *ΣDMAPs* sum of DMP, DMTP, and DMDTP (nmol/L); *ΣDEAPs* sum of DEP, DETP, and DEDTP (nmol/L); *ΣDAP* sum of six DAPs (nmol/L); *LOD* limit of detection; *> LOD* detection rate (%); *GM* geometric mean; *Min.* minimum value; *Max.* maximum value

The ICCs of the six DAPs, ΣDMAP, ΣDEAP, and ΣDAP and those adjusted by Cr concentrations are presented in Table 2. Only the unadjusted single measure ICC for DMDTP was calculated to be less than 0.40, and the other single measure ICCs ranged between 0.41 and 0.51. All average measure ICCs were greater than 0.80 due to multiple measurements. Correction by Cr concentrations did not affect single and average measure ICCs except DMDTP (0.37 vs. 0.46) and DEP (0.46 vs. 0.55). ICCs of FMV were compared with those calculated from the spot urine samples obtained in the same afternoon (Table 3 and Additional file 1: Table S2). Some single measure ICCs of FMV were less than 0.40 (unadjusted DMTP, DMDTP, ΣDMAP, and ΣDAP and Cr-adjusted DMTP and ΣDMAP), whereas all single measure ICCs of the afternoon were over 0.40. The ICCs of Cr-adjusted and unadjusted values in the afternoon were comparable or greater than those of FMV except unadjusted DEP, DEDTP, and ΣDEAP and Cr-adjusted DMDTP and DEDTP. The concentration values of metabolites with low detection rates and with low concentrations tended to vary, which led to lower ICCs. In general, the ICCs of samples taken in the afternoon were better than those of FMV, which were consistent with the results of average ICC measurements (Table 3 and Additional file 1: Table S2).

**Table 2:** Unadjusted and Cr-adjusted ICCs of urinary OP metabolites

Compounds	Unadjusted		Cr-adjusted	
	ICC (1.1)	ICC (1.k)	ICC (1.1)	ICC (1.k)
DMP	0.49	0.91	0.48	0.90
DMTP	0.41	0.87	0.42	0.88
DMDTP	0.37	0.86	0.46	0.89
DEP	0.46	0.90	0.55	0.93
DETP	0.48	0.90	0.49	0.91
DEDTP	0.51	0.91	0.50	0.91
ΣDMAP	0.45	0.89	0.48	0.90
ΣDEAP	0.49	0.91	0.57	0.93
ΣDAP	0.47	0.90	0.53	0.92

*DMP* dimethylphosphate; *DMTP* dimethylthiophosphate; *DMDTP* dimethyldithiophosphate; *DEP* diethylphosphate; *DETP* diethylthiophosphate; *DEDTP* diethyldithiophosphate; *ΣDMAP* sum of DMP, DMTP, and DMDTP; *ΣDEAP* sum of DEP, DETP, and DEDTP; *ΣDAP* sum of six DAPs; *OP* organophosphate insecticides; *Cr* urinary creatinine; *ICC* intraclass correlation coefficient; *ICC (1.1)* single measure ICC; *ICC (1.k)* average measure ICCs

**Table 3:** Cr-adjusted ICCs of urinary OP metabolites affected by time of day of urine sampling

Compounds	ICC (1.1)		ICC (1.k)	
	FMV	PM	FMV	PM
DMP/Cr	0.44	0.52	0.80	0.84
DMTP/Cr	0.33	0.45	0.71	0.80
DMDTP/Cr	0.46	0.41	0.81	0.77
DEP/Cr	0.53	0.55	0.85	0.86
DETP/Cr	0.43	0.49	0.79	0.83
DEDTP/Cr	0.61	0.47	0.89	0.82
ΣDMAP/Cr	0.39	0.50	0.76	0.84
ΣDEAP/Cr	0.53	0.57	0.85	0.87
ΣDAP/Cr	0.45	0.55	0.80	0.86

*DMP* dimethylphosphate; *DMTP* dimethylthiophosphate; *DMDTP* dimethyldithiophosphate; *DEP* diethylphosphate; *DETP* diethylthiophosphate; *DEDTP* diethyldithiophosphate; *ΣDMAP* sum of DMP, DMTP, and DMDTP; *ΣDEAP* sum of DEP, DETP, and DEDTP; *ΣDAP* sum of six DAPs; *OP* organophosphate insecticides; *Cr* urinary creatinine; *ICC* intraclass correlation coefficient; *ICC (1.1)* single measure ICC; *ICC (1.k)* average measure ICCs; *FMV* first morning void; *PM* spot urine in afternoon

The surrogate category analyses were conducted to estimate the frequency of misclassification. Table 4 and Additional file 1: Table S3 indicate that a spot sampling generally resulted in the misclassification of exposure categories at less than 10%. Adjustments of Cr did not affect probability except DMDTP and ΣDAP, which reduced by 17 points (92.5 to 75.4%) and maintained a remarkably high rate (95.3 to 98.9%), respectively. Regarding DMDTP, the extent of misclassification would be smaller when we used spot urine samples, but not FMV samples.

**Table 4:** Surrogate category analyses based on a single random Cr-adjusted sample obtained from a set of 1000 resamples (%)

Compounds	All	FMV	PM
DMP/Cr	97.4	96.3	99.8
DMTP/Cr	95.6	95.6	94.7
DMDTP/Cr	75.4	74.7	87.5
DEP/Cr	90.2	93.6	91.2
DETP/Cr	84.6	86.6	91.4
DEDTP/Cr	93.0	88.1	98.9
ΣDMAP/Cr	97.8	95.5	99.3
ΣDEAP/Cr	90.9	94.2	91.9
ΣDAP/Cr	98.9	98.9	99.5

*DMP* dimethylphosphate; *DMTP* dimethylthiophosphate; *DMDTP* dimethyldithiophosphate; *DEP* diethylphosphate; *DETP* diethylthiophosphate; *DEDTP* diethyldithiophosphate; *ΣDMAP* sum of DMP, DMTP, and DMDTP; *ΣDEAP*, sum of DEP, DETP, and DEDTP; *ΣDAP* sum of six DAPs; *Cr* urinary creatinine; *FMV* first morning void; *PM* spot urine in afternoon

Overall, these results indicated that participants were categorized accurately into four exposure groups according to the quartile points except Cr-adjusted DMDTP.

## Discussion

In this study, six DAP metabolites were measured in ten urine samples among 62 pregnant Japanese women (Additional file 2: Figure S1). To our knowledge, this is the first study on OP insecticide exposure among pregnant women living in Japan. For all DAP metabolites, the Cr-adjusted single ICCs exceeded 0.4, indicating moderate reliability as the biomarker of exposure to OP insecticides. Regarding inter-individual variations, surrogate category analyses showed participants were categorized accurately into four exposure groups according to the quartile points.

In intra-individual variation assays, single measurements using single spot urine samples elicited moderate reliability in the estimation of short-term daily DAP levels in pregnant women who lived in typical urban environments in Japan. Adjustments by Cr level greatly improved ICCs for DMDTP, DEP, ΣDEAP, and ΣDAP. For ΣDMAP, ΣDEAP, and ΣDAP, the estimated ICCs were equal to 0.28, 0.24, and 0.30 in urine samples taken at < 18, 18–25, and > 25 weeks into pregnancy, respectively, in Netherlandic women [12]. ICC values for both uncorrected and specific gravity-corrected DETP and DMTP were approximately equal to 0.2 (95% CI, approximately 0.05–0.47) in pregnant women in Puerto Rico. [13]. Based on Landis and Koch criteria [20] mentioned in the “Methods” section, our ICCs for DAP measurements categorized as “moderate” were considerably higher than the “fair” ICC values previously reported outside Japan. The number of subjects required per group to achieve “almost perfect reliability > 0.8” was from four to ten for FMV and from three to six for urine taken in the afternoon. This discrepancy among the reports was presumably caused by the different sampling periods between the previous studies and our present study. Three-point samples were obtained in 2 months in previous studies [12, 13], while 5-day samples during 2 weeks were obtained in the present study, with some samples taken on consecutive days. Therefore, carryover effects from the preceding day could be expected in the present study owing to the half-lives in the range of 2 to 15.5 h [23, 24]. However, the ICCs in all 5-day samples taken within 1 week (*n* = 12) were lower than samples taken from others (all samples taken between 1 and 2 weeks) in this study, suggesting that the short sampling period did not result in higher ICCs.

The sampling season is another factor that presumably affected the ICC in the current study. In the current study, 1, 7, 34, and 20 of the 62 participants provided their samples in June, August, September, and October, respectively. ICC values for OP metabolites were reportedly larger in the fall through spring than in the summer, which may reflect seasonal variation in food and pesticide uses [22]. Therefore, the estimated ICCs calculated in the current study were supposed to be high.

ICCs of spot urine taken in the afternoon were generally higher than those of FMV in the present study. Among Cr-adjusted metabolites, DMDTP and DEDTP with detection rates lower than 70% and with lower measured values had better ICCs in FMV; in contrast, other metabolites with detection rates higher than 85% had better ICCs in the spot urine samples obtained in the afternoon. This finding is similar to our previous study conducted among 5–6-year-old children in which ICCs for five detected Cr-adjusted DAP values except DEDTP were 0.22–0.62 [25]. Regarding DMDTP and DETP in this study, for detection rates lower than 70%, ICCs for these metabolites in afternoon spot urine samples were quite low compared to those in FMV (afternoon spot urine vs. FMV < 0.01 vs. 0.27, 0.15 vs. 0.49, respectively) (unpublished data). In contrast, ICCs for DMP and DMTP, the concentrations of which are occasionally high, were better in the spot urine than in the FMV samples (0.74 and 0.45, 0.56 and 0.48, respectively). The ICCs for the total exposure indices, such as ΣDMAP, ΣDEAP, and ΣDAP, were also higher, with the exception of ΣDEAP, in afternoon spot urine compared to those in FMV samples (ICCs of afternoon spot urine and FMV were 0.70 and 0.43 for ΣDMAP, 0.47 and 0.53 for ΣDEAP, and 0.71 and 0.52 for ΣDAP, respectively) (unpublished data). These data suggest that spot urine in the afternoon is more suitable than FMV when a single measurement is made for six DAPs.

Surrogate category analyses also supported the finding that spot urine sampled in the afternoon is better than FMV in general. The reason why spot urine sample obtained in the same afternoon is better than that obtained as FMV is still unclear, but these results were similar to the reports regarding 3-phenoxybenzoic acid [26] and arsenic [27]. Further studies are needed to clarify the reason.

A limitation of this study is that intra-individual variations of OP insecticides for longer periods are unclear as urine was only sampled within a 2-week period. However, based on the above, these results suggest that one spot sampling is enough to assess short-term (for a few weeks) exposure in pregnancy accurately. Attenuation bias related to low ICCs should be considered when using DAP levels with comparably low ICC levels as biomarkers of OP exposure.

## Conclusion

This study indicated that a single urine sample obtained in the afternoon may be useful in assessing OP exposure as long as the exposure is categorized into quartiles when conducting epidemiological studies in early to mid-pregnant women in Japan, although multiple and sequential sampling would be preferable over single sampling for improved accuracy.

## Additional files

Additional file 1: **Table S1.** Geometric means and percentile values of Cr-adjusted urinary dialkylphosphate concentrations among pregnant women in Japan (*n* = 62). Table S2. Unadjusted ICCs of urinary OP metabolites affected by time of day of urine sampling. Table S3. Surrogate category analyses based on a single random unadjusted sample obtained from a set of 1000 resamples (%). (DOCX 24 kb)

Additional file 2: **Figure S1.** Urinary concentrations of DAP (nmol/g Cr) on a log10 scale for ten urine samples. Each panel represents an individual participant (*n* = 62). Odd (open circle, ○) and even numbers (filled circle, ●) are first void and afternoon spot urine samples in chronological order, respectively. Repeats 1 and 2, 3 and 4, 5 and 6, 7 and 8, and 9 and 10 were conducted on the same day. (ZIP 7609 kb)

## Abbreviations

Cr: Creatinine
DAPs: Dialkylphosphates
DEDTP: Diethyldithiophosphate
DEP: Diethylphosphate
DETP: Diethylthiophosphate
DMDTP: Dimethyldithiophosphate
DMP: Dimethylphosphate
DMTP: Dimethylthiophosphate
FMV: First morning void
GM: Geometric mean
ICC: Intraclass correlation coefficient
LOD: Limit of detection
Max.: Maximum value
Min.: Minimum value
OP: Organophosphate
PM: Spot urine obtained in the afternoon
UPLC-MS/MS: Ultra performance liquid chromatography with tandem mass spectrometry
ΣDAP: The sum of ΣDMAP and ΣDEAP
ΣDEAP: The sum of DEP, DETP, and DEDTP
ΣDMAP: The sum of DMP, DMTP, and DMDTP
