# Supplementary material for: Intra-individual variations of organophosphate pesticide metabolite concentrations in repeatedly collected urine samples from pregnant women in Japan
Source: Environ Health Prev Med. 2019 Jan 17;24:7. doi: 10.1186/s12199-019-0761-4 (PMC6337762; doi:10.1186/s12199-019-0761-4)
Supplement: Supplementary file 1 — Table S1. Geometric means and percentile values of Cr-adjusted urinary dialkylphosphate concentrations among pregnant women in Japan (n = 62). Table S2. Unadjusted ICCs of urinary OP metabolites affected by time of day of urine sampling. Table S3. Surrogate category analyses based on a single random unadjusted sample obtained from a set of 1000 resamples (%). (DOCX 24 kb) [file 12199_2019_761_MOESM1_ESM.docx]

Additional file 1

Table S1 Geometric means and percentile values of Cr-adjusted urinary dialkylphosphate concentrations among pregnant women in Japan (n=62)

| Compounds | FMV | | | | | | | | |  | | PM | | | | | | | | | Between  -variance | | Within  -variance | |
| --- | --- | --- | --- | --- | --- | --- | --- | --- | --- | --- | --- | --- | --- | --- | --- | --- | --- | --- | --- | --- | --- | --- | --- | --- |
|  | GM | Min. | Percentile | | | | | Max. |  | | GM | | Min. | Percentile | | | | | Max. |  | |  | |  |
|  |  |  | 5th | 25th | 50th | 75th | 95th |  |  | |  | |  | 5th | 25th | 50th | 75th | 95th |  |  | |  | |  |
| DMP/Cr | 2.0 | <LOD | 0.11 | 0.96 | 2.7 | 5.8 | 19 | 55 |  | | 1.8 | | <LOD | 0.11 | 0.80 | 2.2 | 5.5 | 18 | 98 | 13 | | 1.4 | |  |
| DMTP/Cr | 8.1 | <LOD | 0.78 | 3.2 | 8.1 | 22 | 75 | 262 |  | | 6.8 | | <LOD | 0.55 | 2.4 | 7.3 | 20 | 79 | 569 | 9.7 | | 1.4 | |  |
| DMDTP/Cr | 1.2 | <LOD | <LOD | <LOD | 1.0 | 2.3 | 6.1 | 17 |  | | 1.2 | | <LOD | <LOD | <LOD | 1.0 | 2.5 | 6.7 | 17 | 3.8 | | 0.46 | |  |
| DEP/Cr | 5.0 | <LOD | 1.4 | 2.9 | 4.7 | 8.0 | 22 | 143 |  | | 5.1 | | 0.55 | 1.3 | 2.9 | 4.8 | 8.7 | 23 | 130 | 4.7 | | 0.38 | |  |
| DETP/Cr | 1.0 | 0.11 | 0.22 | 0.43 | 0.80 | 2.2 | 10 | 240 |  | | 1.0 | | 0.11 | 0.22 | 0.41 | 0.82 | 2.2 | 8.7 | 207 | 7.4 | | 0.79 | |  |
| DEDTP/Cr | 0.05 | <LOD | <LOD | <LOD | 0.05 | 0.14 | 0.42 | 1.6 |  | | 0.07 | | <LOD | <LOD | <LOD | 0.07 | 0.18 | 0.67 | 6.9 | 9.0 | | 0.90 | |  |
|  |  |  |  |  |  |  |  |  |  | |  | |  |  |  |  |  |  |  |  | |  | |  |
| ΣDMAPs/Cr | 102 | 4.7 | 16 | 45 | 88 | 237 | 678 | 2139 |  | | 89 | | 4.7 | 12 | 39 | 79 | 217 | 698 | 4500 | 6.7 | | 0.75 | |  |
| ΣDEAPs/Cr | 42 | 3.8 | 11 | 23 | 37 | 66 | 194 | 1963 |  | | 43 | | 5.2 | 11 | 23 | 38 | 72 | 211 | 1521 | 5.0 | | 0.38 | |  |
| ΣDAP/Cr | 158 | 16.0 | 36 | 74 | 140 | 323 | 807 | 4102 |  | | 147 | | 15 | 33 | 69 | 131 | 308 | 818 | 4708 | 5.6 | | 0.49 | |  |

DMP, dimethylphosphate (μg/g); DMTP, dimethylthiophosphate (μg/g); DMDTP, dimethyldithiophosphate (μg/g); DEP, diethylphosphate (μg/g); DETP, diethylthiophosphate (μg/g); DEDTP, diethyldithiophosphate (μg/g); ΣDMAPs, sum of DMP, DMTP, and DMDTP (nmol/g);

ΣDETPs, sum of DEP, DETP, and DEDTP (nmol/g); ΣDAP, sum of six DAP (nmol/g); Cr, creatinine; GM, geometric mean; Min., minimum value; Max., maximum value; FMV, first morning void; PM, spot urine in afternoon

Table S2 Unadjusted ICCs of urinary OP metabolites affected by time of day of urine sampling

| Compounds | ICC (1.1) | |  | ICC (1.k) | |
| --- | --- | --- | --- | --- | --- |
|  | FMV | PM |  | FMV | PM |
| DMP | 0.47 | 0.53 |  | 0.81 | 0.85 |
| DMTP | 0.29 | 0.48 |  | 0.67 | 0.82 |
| DMDTP | 0.31 | 0.40 |  | 0.69 | 0.77 |
| DEP | 0.50 | 0.43 |  | 0.83 | 0.79 |
| DETP | 0.42 | 0.50 |  | 0.78 | 0.83 |
| DEDTP | 0.61 | 0.45 |  | 0.89 | 0.81 |
|  |  |  |  |  |  |
| ΣDMAP | 0.34 | 0.52 |  | 0.72 | 0.84 |
| ΣDEAP | 0.50 | 0.47 |  | 0.83 | 0.82 |
| ΣDAP | 0.39 | 0.52 |  | 0.76 | 0.84 |

DMP, dimethylphosphate; DMTP, dimethylthiophosphate; DMDTP, dimethyldithiophosphate; DEP, diethylphosphate; DETP, diethylthiophosphate; DEDTP, diethyldithiophosphate; ΣDMAPs, sum of DMP, DMTP, and DMDTP; ΣDETPs, sum of DEP, DETP, and DEDTP; ΣDAP, sum of six DAP; OP, organophosphate insecticides; ICC, Intraclass correlation coefficient; ICC (1.1), single measure ICC; ICC (1.k), average measure ICCs; FMV, first morning void; PM, spot urine in afternoon

Table S3 Surrogate category analyses based on a single random unadjusted sample obtained from a set of 1,000 resamples (%)

| Compounds | All | FMV | PM |
| --- | --- | --- | --- |
| DMP | 94.0 | 96.8 | 98.4 |
| DMTP | 93.8 | 94.9 | 95.9 |
| DMDTP | 92.5 | 90.5 | 93.8 |
| DEP | 89.0 | 98.8 | 93.2 |
| DETP | 89.8 | 88.4 | 95.0 |
| DEDTP | 95.8 | 84.2 | 96.2 |
|  |  |  |  |
| ΣDMAP | 93.9 | 95.6 | 98.2 |
| ΣDEAP | 94.3 | 97.4 | 94.0 |
| ΣDAP | 95.3 | 94.6 | 98.9 |

DMP, dimethylphosphate; DMTP, dimethylthiophosphate; DMDTP, dimethyldithiophosphate;

DEP, diethylphosphate; DETP, diethylthiophosphate; DEDTP, diethyldithiophosphate;

ΣDMAPs, sum of DMP, DMTP, and DMDTP; ΣDETPs, sum of DEP, DETP, and DEDTP;

ΣDAP, sum of six DAP; FMV, first morning void; PM, spot urine in afternoon
