# Supplementary figures and images for: Intra-individual variations of organophosphate pesticide metabolite concentrations in repeatedly collected urine samples from pregnant women in Japan
Source: Environ Health Prev Med. 2019 Jan 17;24:7. doi: 10.1186/s12199-019-0761-4 (PMC6337762; doi:10.1186/s12199-019-0761-4)

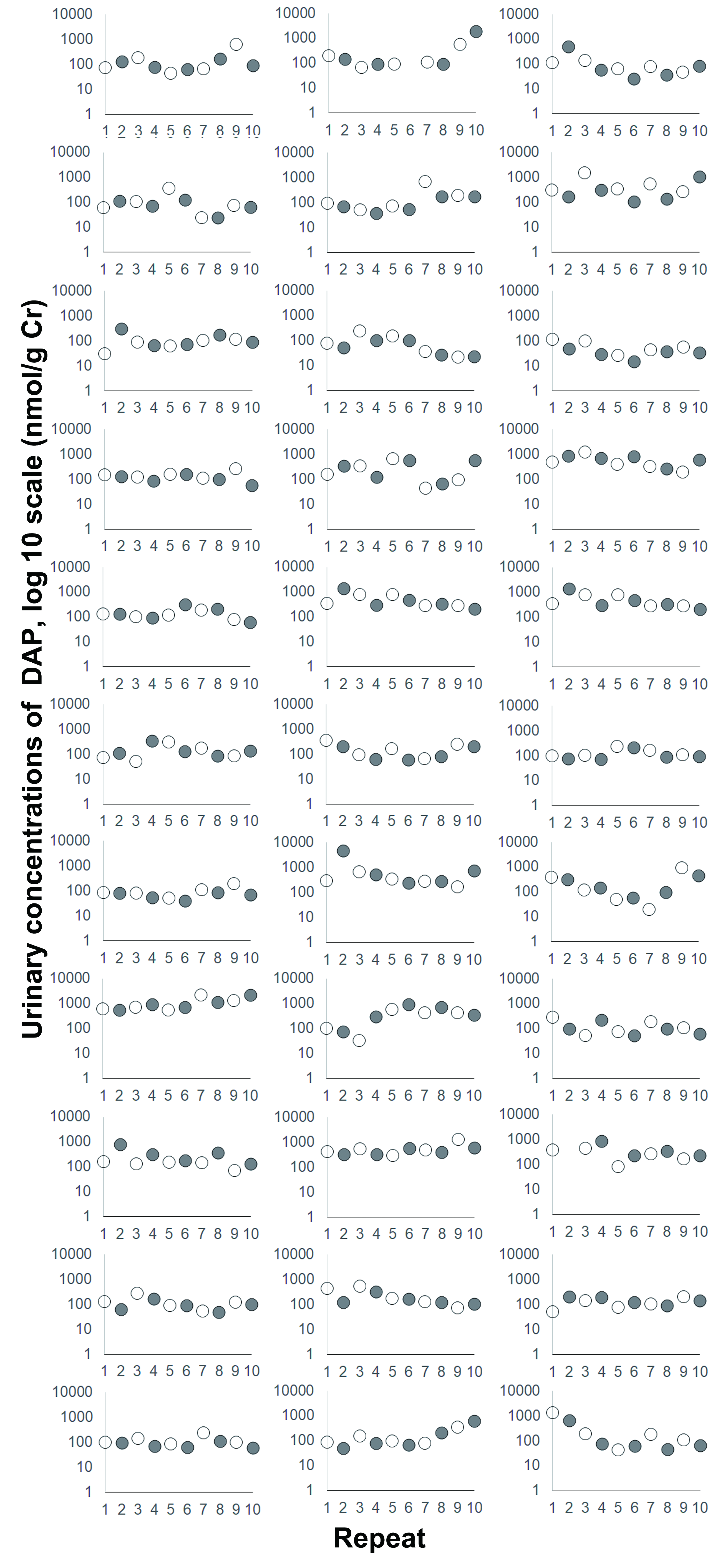

Supplement: Supplementary file 2 — Figure S1. Urinary concentrations of DAP (nmol/g Cr) on a log10 scale for ten urine samples. Each panel represents an individual participant (n = 62). Odd (open circle, ○) and even numbers (filled circle, ●) are first void and afternoon spot urine samples in chronological order, respectively. Repeats 1 and 2, 3 and 4, 5 and 6, 7 and 8, and 9 and 10 were conducted on the same day. (ZIP 7609 kb) [file 12199_2019_761_MOESM2_ESM.zip › Supplementary Fig1-1.tif]

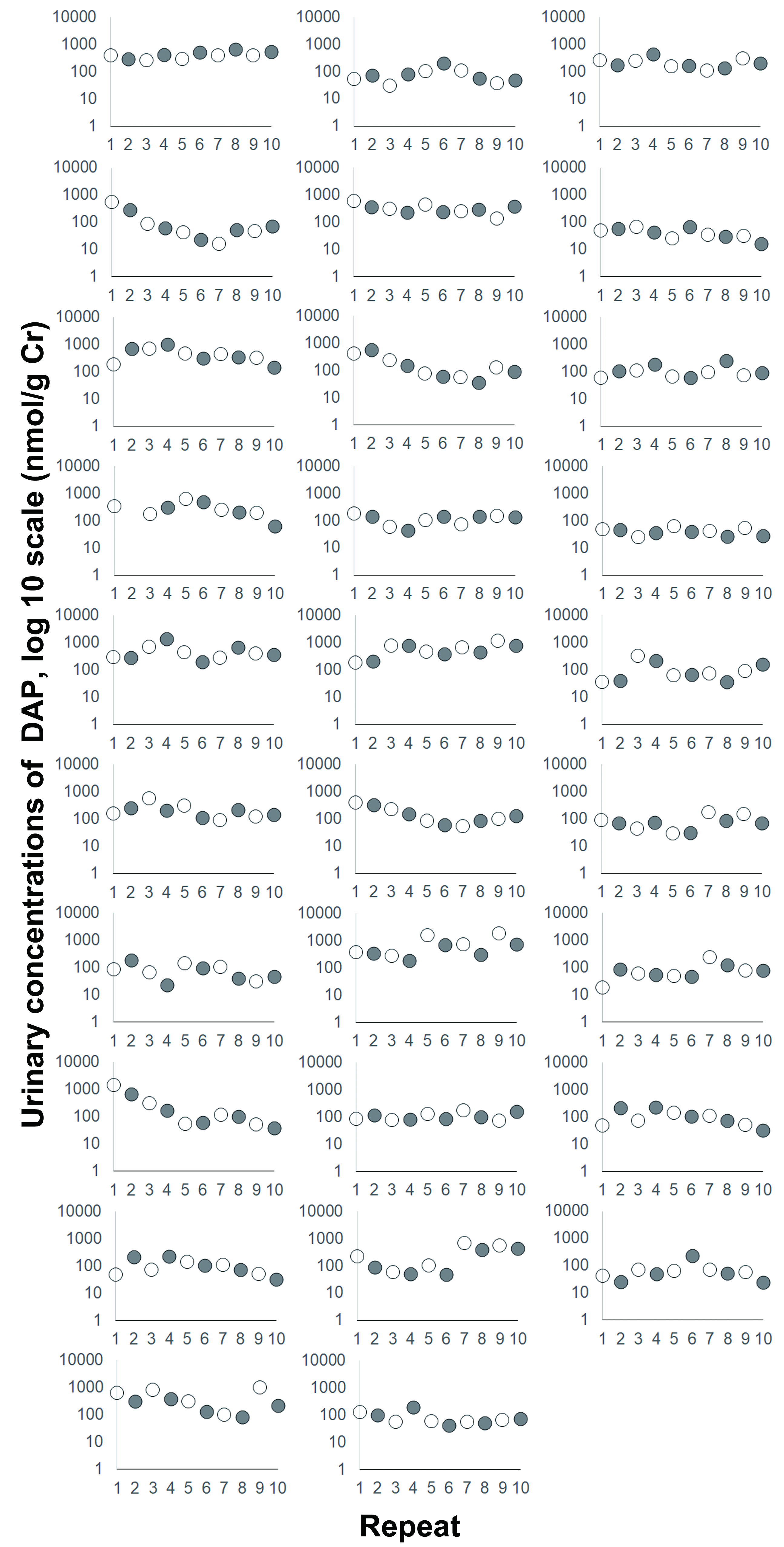

Supplement: Supplementary file 2 — Figure S1. Urinary concentrations of DAP (nmol/g Cr) on a log10 scale for ten urine samples. Each panel represents an individual participant (n = 62). Odd (open circle, ○) and even numbers (filled circle, ●) are first void and afternoon spot urine samples in chronological order, respectively. Repeats 1 and 2, 3 and 4, 5 and 6, 7 and 8, and 9 and 10 were conducted on the same day. (ZIP 7609 kb) [file 12199_2019_761_MOESM2_ESM.zip › Supplementary Fig1-2.tif]
